# Extrusion of Cell Encapsulated in Boron Nitride Nanotubes Reinforced Gelatin—Alginate Bioink for 3D Bioprinting

**DOI:** 10.3390/gels8100603

**Published:** 2022-09-21

**Authors:** Akesh Babu Kakarla, Ing Kong, Cin Kong, Helen Irving, Colleen J. Thomas

**Affiliations:** 1 School of Computing, Engineering and Mathematical Sciences, La Trobe University, Bendigo, VIC 3552, Australia; 2 Department of Biomedical Sciences, Faculty of Science and Engineering, University of Nottingham Malaysia Campus, Semenyih 43500, Selangor, Malaysia; 3 Department of Rural Clinical Sciences, La Trobe Institute for Molecular Sciences (LIMS), Bendigo, VIC 3552, Australia; 4 Department of Microbiology, Anatomy, Physiology and Pharmacology, School of Agriculture, Biomedicine and Environment, La Trobe University, Melbourne, VIC 3086, Australia; 5 Centre for Cardiovascular Biology and Disease Research, School of Agriculture, Biomedicine and Environment, La Trobe University, Melbourne, VIC 3086, Australia; 6 Pre-Clinical Critical Care Unit, Florey Institute of Neuroscience and Mental Health, University of Melbourne, Melbourne, VIC 3052, Australia

**Keywords:** bioprinting, alginate, gelatin, boron nitride nanotube, hydrogels, bioinks, THP-1

## Abstract

Three-dimensional (3D) bioprinting, an innovative technology, has gained the attention of researchers as a promising technique for the redevelopment of complex tissue or organ structures. Despite significant advancements, a major challenge in 3D bioprinting is the limited number of suitable bioinks that fulfil the physiochemical requirements to produce complicated structures. Therefore, there is a demand for the production of bioinks for 3D bioprinting techniques. In this short communication, THP-1 cells encapsulated in boron nitride nanotubes (BNNTs) reinforced gelatin and alginate bioink was prepared. The study investigated the impact on the cells during printing using a fluorescence cell image. The results showed that the pure polymer bioinks demonstrated poor printability properties with the incorporation of cells. However, BNNT-combined bioink showed a significant increase in structural integrity even after the incorporation of cells. Furthermore, the scaffold structure was successfully printed with the cells incorporated bioink, and a considerable number of live cells were observed. With further studies, BNNTs as a promising nanomaterial for formulating bioink encapsulated with cells can be understood fully.

## 1. Introduction

Three-dimensional (3D) bioprinting has emerged as a new hope in tissue engineering applications in regenerative medicine to develop complex 3D structures [1,2,3]. The technology involves depositing a substance composed of various cells and biomaterials in a layer-by-layer format to generate biomedical structures [4,5,6]. The substance used in bioprinting is referred to as bioink [7]. Hydrogels have been recommended as an ideal matter for bioinks formulation [8,9]. Hydrogels contain high water levels, are highly biocompatible and resemble extracellular matrix (ECM), making them efficient bioinks for 3D bioprinting [9,10]. However, employing hydrogels in the bioprinting process is challenging because it requires suitable mechanical and gelation properties to maintain a constant deposition from the printer and to develop rigid structures [11]. Furthermore, another major issue in maintaining good cell viability and cell encapsulation after printing is to produce a stable structure and facilitate the growth of tissue or organ microbiological environments [12]. Due to these challenges, improvements in a printable hydrogel that can support stable structure and support cell functioning are needed to accelerate current techniques in tissue engineering applications [7,13]. 

Several types of hydrogels have been formulated as bioink to address this challenge. Among them, natural hydrogels prepared with gelatin [14,15,16] and alginate (Alg) [17] were widely studied as bioinks, cell encapsulates and scaffolds for tissue engineering. Gelatin can provide a micro-environment to improve cell adhesions and proliferation [14], while Alg has the characteristics of rapid gelation through crosslinking with calcium ions (Ca^2+^) [18]. In terms of gelatin, Leucht et al. [14] showed the bioprinting of vascularised bone equivalents using pure gelatin and gelatin modified with methacrylate and acetylate-based bioinks. The results stated that modified gelatin bioinks displayed good printability and higher swelling properties than pure gelatin. Moreover, the printed vascularised structures indicated adequate support for osteogenic differentiation of dermal microvascular endothelial cells (HDMECs) and human adipose-derived stem cells (ASCs) after printing.

Similarly, Jia et al. [17] investigated the effects of viscosity and density of Alg bioink for bioprinting. The study indicated that Alg encapsulated with human ASCs could produce high-resolution printing structures [17]. Furthermore, it was also stated that the tuneable properties of Alg, such as density and viscosity, allowed higher cell viability after printing [17]. However, single-component hydrogels were significantly limited in providing good mechanical properties and impacting cell behaviour within printed constructs. Therefore, researchers formulated a combination of two materials to produce bioinks. Chung et al. [19] studied the printability of gelatin-Alg bioink for extrusion printing of cells. The study stated that the mechanical properties of gelatin-Alg decreased by more than 60% in cell culture media [19]. However, optimum printability was achieved by controlling temperature and higher viscosity [19]. In another study, Giuseppe et al. [20] investigated the printability of various concentrations of gelatin-Alg for bioprinting applications. The results demonstrated that gelatin 7% w × v^−1^ and Alg 8% w × v^−1^ had good printability and mechanical properties [20]. Similarly, Li et al. [18] reported the mechanical and printability properties of gelatin-Alg bioink encapsulated stem cells for extrusion bioprinting. After printing, the gelatin-Alg bioink displayed good cell proliferation and differentiation [18]. 

Recently, nanocomposite bioinks have gained increasing attention from researchers to enhance the physicochemical and structural stability of 3D bioprinted models [21]. Luo et al. [22] investigated the printability of gelatin-Alg reinforced with cellulose nanofibre (CNF) bioink for the bioprinting of meniscal tissues. The results showed higher rheological performance and printability with incorporating CNF into pure gelatin-alginate bioink. In addition, gelatin-Alg reinforced with CNF has maintained a reasonable cell viability rate. Likewise, Li et al. [23] prepared gelatin-Alg reinforced with carbon nanotubes (CNTs) to develop a bioink for 3D bioprinted blood vessels. The results showed that incorporating CNTs effectively increased the mechanical properties with minor cytotoxicity [23]. In another study, Li et al. [24] reinforced gelatin-Alg bioink with bioactive nanoparticles (BNPs) that released silicon ions to maintain mesenchymal stem cells (MSCs). It was stated that BNP-reinforced gelatin-Alg bioink showed high printability and the ability to maintain MSCs cells without adverse effects [24]. Another noteworthy nanomaterial is boron nitride nanotubes (BNNTs). BNNTs possess unique chemical, mechanical, and electrical properties as one-dimensional nanomaterial. In addition, BNNTs could promote cell proliferation and differentiation without any adverse toxic effects. For instance, Lahiri et al. [25] developed BNNT-reinforced polylactide-polycaprolactone for orthopaedic scaffold applications. The findings revealed that the addition of BNNTs into a polymer matrix increased the mechanical properties and cell viability rate compared to pure polymer [25]. In another study, BNNT-reinforced polycaprolactone filaments were developed for 3D printing for heat dissipation device applications. The 3D-printed radiator models displayed considerable heat dissipation. Thus far, BNNTs-reinforced polymer scaffolds have been prepared using electrospinning, solvent casting and 3D printing for biomedical applications. However, only a few studies have reported BNNTs-reinforced polymers in 3D bioprinting. Therefore, in this study, BNNT-reinforced gelatin-Alg encapsulated THP-1 cells bioink was developed to analyse the cell performance after extrusion.

## 2. Results and Discussion

**Figure 1:**
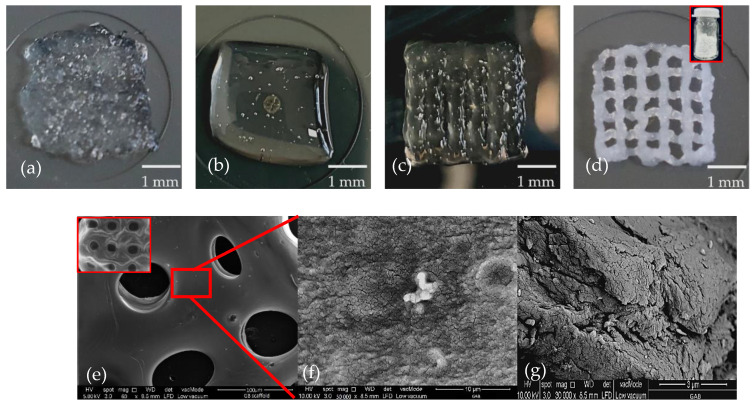

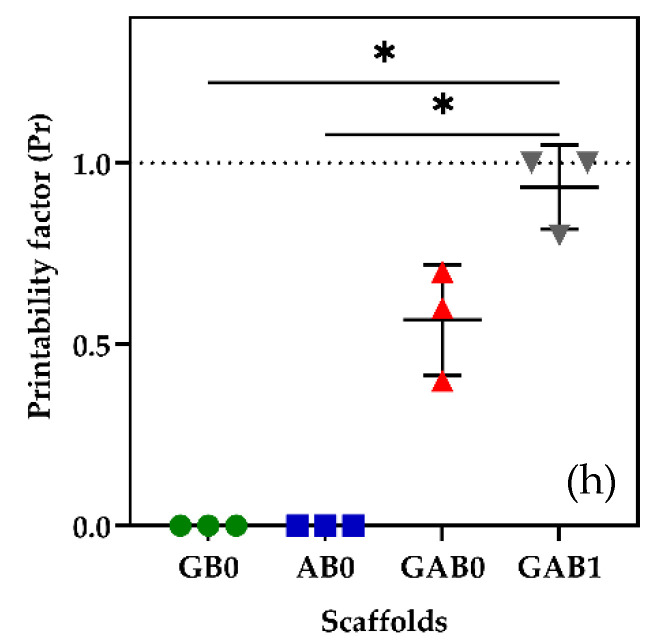
3D bioprinted scaffolds (**a**) GB0; (**b**) AB0; (**c**) GAB0; (**d**) GAB1 (inset BNNTs powder); (**e**) SEM images of GAB1 scaffold at low magnification (inset—GAB1 scaffold topography); (**f**) SEM image of GAB1 scaffold at high magnification; (**g**) cross-sectional view of GAB1 scaffold; (**h**) printability factor of cells incorporated printed scaffolds (*n* = 3, * *p* < 0.05).

Prepared inks such as GB0, AB0, GAB0 and GAB1 were successfully extruded to produce the desired shape (Figure 1a–d). However, as shown in Figure 1a,b, single-component hydrogel inks encapsulated with bioinks do not build gird structures due to a decrease in the viscosity of the material. GAB0 (Figure 1c) showed the slightest improvement in producing the strands but was limited in structural stability. The strand deposition and structural stability were significantly improved with the incorporation of BNNTs into the gelatin and Alg (Figure 1d). The appearance of the GB0, AB0 and GAB0 was brownish, while BNNTs-incorporated polymer hydrogel was white due to uniformly dispersed BNNTs particles. The dispersion of BNNTs was further confirmed with an SEM image as shown in Figure 1e and the inset (Figure 1e) of GAB1 shows good interconnectivity of strands and pore size. The SEM image of GAB1 scaffolds revealed the structure of the strands combined with BNNTs. The topographical view at lower magnification showed well-dispersed BNNTs in the hydrogel network (Figure 1e). While at high magnification, dense chain networks in the submicrometric range were observed (Figure 1f). According to Marmorat et al. [26] fully hydrated gelatin network displayed microstructures similar to the network of mesh or various chains depending on cross-linking density. The higher crosslinking density showed the single strands of gelatin while the low crosslinking density exhibited dense complex strands. Furthermore, Figure 1g shows the cross-sectional of the GAB1 scaffold and evidently displayed an interconnected network in the scaffold which supports the growth of the cell tissue. 

Compared to previous results reported by Kakarla et al. [27] without encapsulation of cells, it was evident that cell density drastically changed the printability of pure polymers compared to the BNNT-incorporated polymer hydrogel bioinks. The printability factor of the scaffolds printed with cells was shown in Figure 1h. It was observed that pure polymer scaffolds could not produce strands. In contrast, the combination of both polymers is able to produce strands with pores. However, the pores were not interconnected as a designed model, especially at the edges of the scaffolds. In addition, GAB1 hydrogel ink produced well-interconnected strands with adequate pore size. According to Avila et al. [26], adding nanofibrillated cellulose to Alg enhanced printability and shape fidelity due to the highly viscous and shear-thinning multicomponent bioink. Similarly, Heggest et al. [28] reported that decreasing the nanocomposite concentration (3.5 wt.%) reduced the shape fidelity of the printed scaffolds.

**Figure 2:**
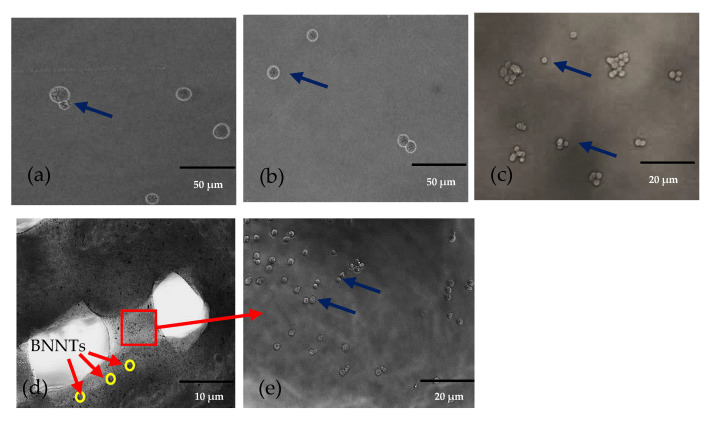
Phase contrast microscopic images of cells in the scaffolds (**a**) GB0; (**b**) AB0; (**c**) GAB0; (**d**) GAB1 at lower magnification (yellow circles-BNNTs); (**e**) GAB1 with cells at higher magnification (cells as indicated by blue arrows).

Figure 2a,b display the fluorescence images of the scaffolds printed with cells. The top surface images show that cells were successfully encapsulated in the hydrogel and extruded through the nozzle with continuous flow. However, a higher density of cells in the hydrogel could block the nozzles that affect the continuous extrusion of the bioink [29]. Thus, the cell density was kept to 2.5 × 10^5^ cells × mL^−1^ in this study. The lower magnification formulated bioink scaffold images displayed in Figure 2a–d revealed that cells were covered by hydrogels extruded without causing adverse effects to the cells. Figure 2d shows the topology of the 3D bioprinted structure of GAB1 incorporated with cells, while the black colour particles (yellow circle) in the GAB1 were BNNTs. The image at higher magnification shows the cells integrated into the bioink strands. Thus, it was evident that cells were extruded with a continuous flow along with the bioink material. 

**Figure 3:**
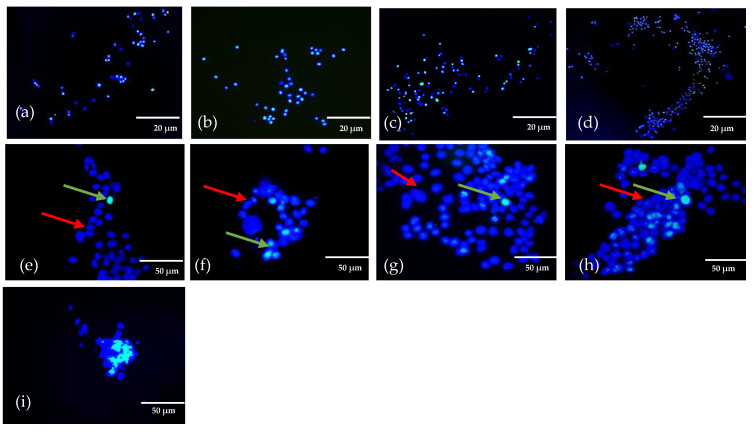
Scaffolds treated with live/dead cell reagents at lower magnification of live/dead cells (**a**) GB0; (**b**) AB0; (**c**) GAB0; (**d**) GAB1; higher magnification of live/dead cells (**e**) GB0; (**f**) AB0; (**g**) GAB0; (**h**) GAB1; (**i**) higher magnification in GAB1 cell agglomeration. (Red arrow represents live cells and green arrow represents dead cells).

The bioink extruded with cells must show good cell combability because the structures used to grow anatomical models for biomedical applications. Live/dead staining was used to examine the effect of cell survival rate after extrusion. Live/dead analysis showed (Figure 3a–i) the live cells in red arrow and dead or apoptosis cells in green colour. Figure 3a–d shows the cells at lower magnification. Figure 3e–h show images of 3d bioprinted scaffolds of bioink encapsulated with cells at higher magnification. The fluorescence images displayed uniform distribution of the cells after extrusion. Furthermore, the results validated that the extrusion method associated with shear force to produce the scaffolds in a layer-by-layer format does not damage cells. Schwartz et al. [30] demonstrated the cells encapsulated gelatin bioink and the impacts on cells while 3D printing. The study stated that extrusion pressure and steady shear viscosity play a vital role in cell viability after extrusion to produce an adequate resolution of bioprinted constructs. Moreover, agglomerated cells are clustered during printing in GAB1 after extrusion, as shown in Figure 3i. Bhattacharya et al. [31] reported that bioink homogeneity in osteoblast cells mixed with alpha-tricalcium phosphate bioink. The results stated that agglomerated cells as clustered could be expected due to cell coverage around the nanomaterials. Besides, it was stated that differentiating between cells and bioink matrix could be difficult due to the autofluorescence imparted by the bioink [11,30,32,33]. Nevertheless, it can be observed that live cells embedded with printed bioink produced the desired structures at lower magnification. GAB1 displayed optimal printability with better structural integrity encapsulated with cells than pure polymer bioink.

## 3. Conclusions

Preliminary studies with cell-encapsulated bioink composed of gelatin, Alg and BNNTs were successfully extruded, and cell viability was investigated after extrusion. It is worth mentioning that cell density decreased the printability of pure polymers compared to the BNNT-incorporated polymer bioinks. The current study highlights cell imaging after printing using live/dead reagents. It was observed that cells were successfully embedded in the bioink and extruded. However, the present study limited cell imaging analysis by investigating a single cell type and density. Thus, different cell densities need to be explored further to understand the impact on the printability of GAB1. In addition, it is also necessary to study the cell DNA damage after extrusion through polymerase chain reaction and gene expression to better understand the cell structures after extrusion under mechanical force.

## 4. Materials and Methodology

### 4.1. Materials

Gelatin, alginate (medium viscosity), calcium chloride (CaCl_2_), and phosphate-buffered solution (PBS) pH 7.4 were purchased from Sigma Aldrich, Melbourne, Australia. Roswell Park Memorial Institute (RPMI) 1640 medium, foetal bovine serum (FBS), Invitrogen™-ready probes for live and dead cell stains and propidium iodonium were purchased from Sigma Aldrich, Melbourne, Australia. THP-1 cells were acquired from Cell Bank, Westmead, Australia. BNNTs were produced through co-precipitation and annealing [34,35] and functionalised with hydroxyl groups [36]. 

### 4.2. Preparation of Nanocomposite Bioink

**Figure 4:**
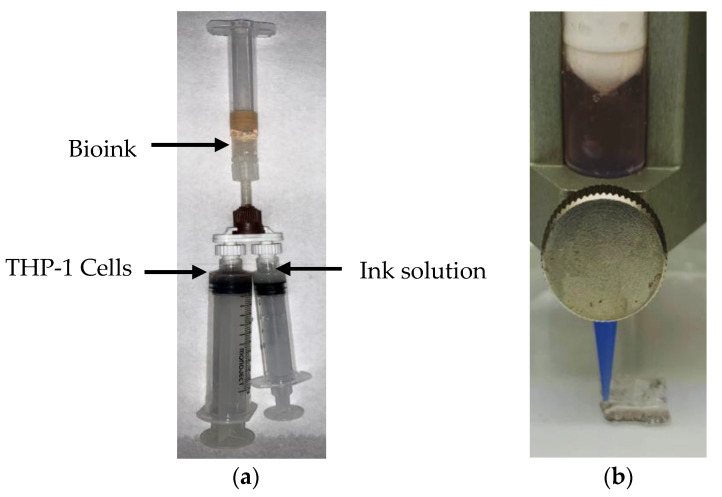
(**a**) Mixing hydrogels with cells using cell mixer; (**b**) 3D bioprinting of bioink.

According to our previous reports [27,35], hydrogel composite bioink was initially created from gelatin, Alg and BNNTs. Briefly, gelatin (6 w × v^−^^1^%) was first mixed with deionised water and Alg (5 w × v^−^^1^%) was added to the same solution through vigorous stirring. Afterwards, the BNNTs (0.1 w × v^−^^1^%) were added to the gelatin-Alg combination to obtain nanocomposite hydrogel suspension. Finally, THP-1 cells of density 2.5 × 10^5^ cells × mL^−1^ were added to the nanocomposite hydrogel suspension to get nanocomposite bioink using a cell mixer, as shown in Figure 4a. Formulated bioinks are shown in Table 1; they are abbreviated as GB0, AB0, GAB0 and GAB1.

### 4.3. 3D Bioprinting of Nanocomposite Bioink

The bioinks were printed in grid-like structures with 10 mm in length, width of 10 mm and height of 1 mm (Figure 4b) using Cellink INKREDIBLE+ (Cellink, Goteborg, Sweden). The bioink was drawn into a 3 mL syringe attached to a 22-gauge nozzle. Later, the bioink was extruded under constant pressure, as shown in Table 1, onto a Petri dish at room temperature (see the Supplementary Materials). The obtained scaffolds were crosslinked with 2.5% GTA for pure gelatin and 100 mM of CaCl_2_ for pure alginate, GAB0 and GAB1 scaffolds immediately after printing for 10 min. Afterwards, the scaffolds were washed with PBS two times. 

### 4.4. Morphology

The morphology of the scaffolds was examined using a scanning electron microscope (FESEM, Hitachi SU7000, Tokyo, Japan) [26]. The images were obtained at lower vacuum mode at a voltage of 5 to 10 kV.

### 4.5. Printability

After bioprinting, the scaffold images were taken using a 12-megapixel camera to measure the printability. The printability factor (Pr) of the 3D bioprinted scaffolds was measured according to reports suggested by Ouyang et al. [12] and Habib et al. [28]. The scaffolds were printed with a square shape pore. The data were reported using a mean of three replicates using GraphPad Prism software (V9.0, San Diego, CA, USA). The one-way analysis of variances for normal distribution was used to obtain statistical significance. ∗ *p* < 0.05 was significant variation.

(1) Printability factor Pr=L216A

where *L* and *A* represent the length and area of the outcome pores. The pore with a uniform square with good pore interconnectivity significances Pr = 1. Pr less than 1 indicates the lesser poor interconnectivity without considerable strands.

### 4.6. Cell Viability of 3D Bioprinted Structures

After bioprinting, the scaffolds Live/dead staining was used to examine the cell viability. The printed scaffolds with cells were dyed with Ready Probes™ Cell Viability Imaging Kit (blue/green) (Invitrogen™). Stained cells were imaged using fluorescence microscopy (BX71, Olympus, Tokyo, Japan) to identify the apoptosis and live cells.

## Figures and Tables

**Table 1:** Formulated bioinks and parameters for extrusion bioprinting.

Ink Solution	Gelatin (G) (w × v^−1^%)	Alginate (A) (w × v^−1^%)	BNNTs (B) (w × v^−1^%)	Nozzle Gauge (G)	Inner Diameter (mm)	Pressure (kPa)	THP-1 Cell Density Cells × mL^−1^	Crosslinking
GB0	6	0	0	22	0.41	25 ± 1	2.5 × 10^5^	GTA
AB0	0	5	0	22	0.41	25 ± 2	2.5 × 10^5^	CaCl_2_
GAB0	6	5	0	22	0.41	30 ± 2	2.5 × 10^5^	CaCl_2_
GAB1	6	5	0.1	22	0.41	50 ± 2	2.5 × 10^5^	CaCl_2_

## Data Availability

Not applicable.

## Supplementary Materials

The following supporting information can be downloaded at: https://www.mdpi.com/article/10.3390/gels8100603/s1, Video S1.
